# Abnormal cerebellar volume in somatic vs. non-somatic delusional disorders

**DOI:** 10.1186/s40673-020-0111-8

**Published:** 2020-01-20

**Authors:** Joshua Krämer, Markus Huber, Christina Mundinger, Mike M. Schmitgen, Roger Pycha, Erwin Kirchler, Christian Macina, Martin Karner, Dusan Hirjak, Katharina M. Kubera, Malte S. Depping, Dmitry Romanov, Roland W. Freudenmann, Robert Christian Wolf

**Affiliations:** 1 Department of General Psychiatry, Center for Psychosocial Medicine, Heidelberg University, Heidelberg, Germany; 2 Department of Psychiatry, General Hospital Bruneck, Bruneck, South Tyrol Italy; 3 Department of Radiology, General Hospital Bruneck, Bruneck, South Tyrol Italy; 4 Department of Psychiatry and Psychotherapy, Central Institute of Mental Health, Medical Faculty Mannheim, Heidelberg University, Mannheim, Germany; 5 Department of Psychiatry and Psychosomatics, I.M. Sechenov First Moscow State Medical University, Moscow, Russia; 6 Department of Psychiatry and Psychotherapy III, Ulm University, Ulm, Germany

**Keywords:** Cerebellum, Delusions, Paranoid, Delusional infestation, Voxel-based morphometry, SUIT

## Abstract

**Background:**

There is abundant evidence for cerebellar involvement in schizophrenia, where the cerebellum has been suggested to contribute to cognitive, affective and motor dysfunction. More recently, specific cerebellar regions have also been associated with psychotic symptoms, particularly with auditory verbal hallucinations. In contrast, little is known about cerebellar contributions to delusions, and even less is known about whether cerebellar involvement differs by delusional content.

**Methods:**

Using structural magnetic resonance imaging at 1.0 T together with cerebellum-optimized segmentation techniques, we investigated gray matter volume (GMV) in 14 patients with somatic-type delusional disorder (S-DD), 18 patients with non-somatic delusional disorder (NS-DD) and 18 patients with schizophrenia (SZ) with persistent non-somatic delusions. A total of 32 healthy controls (HC) were included. Between-group comparisons were adjusted for age, gender, chlorpromazine equivalents and illness duration.

**Results:**

Compared to HC, S-DD patients showed decreased GMV in left lobule VIIIa. In addition, S-DD patients showed decreased GMV in lobule V and increased GMV in bilateral lobule VIIa/crus II compared to NS-DD. Patients with SZ showed increased GMV in right lobule VI and VIIa/crus I compared to HC. Significant differences between HC and NS-DD were not found.

**Conclusions:**

The data support the notion of cerebellar dysfunction in psychotic disorders. Distinct cerebellar deficits, predominantly linked to sensorimotor processing, may be detected in delusional disorders presenting with predominantly somatic content.

## Background

There is abundant evidence that the cerebellum subserves a variety of cognitive functions, including sensorimotor integration, executive control, self-reflection and emotional regulation [1–5]. In particular, it seems to play an important role in many perceptual processes, including processing and prediction of somatosensory input [6].

In schizophrenia (SZ), cerebellar abnormalities have been observed in terms of brain structure and function [7–10]. The majority of functional studies focused on changes in the cortico-cerebellar-thalamic-cortical circuit (CCTCC) as put forward by the concept of “cognitive dysmetria”, e. g. [11–15]. This model suggests that cognitive abilities, similar to motor functions, are supported by a CCTCC feedback loop that normally monitors and controls mental activity. CCTCC disruption is thought to be associated with cognitive dysfunction in SZ, i. e. with difficulties in retrieving, prioritizing, processing, coordinating and expressing information, which in turn might explain the broad variety of symptoms, including hallucinations and delusions [9, 16]. More recently, auditory verbal hallucinations (AVH) in SZ have been associated with lower gray matter volume (GMV) in specific cerebellar regions [17] and with aberrant functional connectivity of the cerebellum [18]. At present, very little is known about specific cerebellar contributions to delusions. Several case reports have linked cerebellar lesions, i. e. stroke and neoplasia, with the development of delusions [19–22]. However, we are not aware of any studies explicitly investigating contributions of specific cerebellar subregions to delusions in psychotic disorders.

Delusions can be related to a vast range of diverse topics. In particular, recurring themes in delusional disorders comprise erotomania, grandiosity, jealousy, persecution and somatic disease (somatic-type delusional disorder [S-DD] according to DSM-5). A typical form of S-DD is delusional infestation (DI), also known as delusional parasitosis or Ekbom syndrome. Affected patients have the fixed belief that small living or (much rarer) inanimate pathogens such as insects or worms infest their body, although there is no medical evidence of their presence [23, 24]. Those beliefs are typically accompanied by related tactile sensations of crawling, stinging or biting on the skin or elsewhere in the body [25]. It is often unclear if those sensations qualify as hallucinations or merely as illusions [23].

Several cognitive-behavioral models of delusion formation have been proposed in the past, with accompanying predictions of related neural correlates [26]. The so-called “two-factor model of delusions” postulates that two factors are necessary for the emergence of delusions: While a first factor is responsible for the emergence of delusional ideas, a second factor is responsible for impaired reasoning and thus for the failure to reject those ideas [27]. In line with the notion of a content-specific first factor, several studies have associated distinct delusional contents with specific neural correlates. For instance, in patients with SZ-spectrum disorders presenting with prominent paranoid and persecutory delusions, structural and functional imaging studies have identified alterations in neural systems involved in threat and fear processing (including the amygdala and cingulate cortex) [28–30]. In patients with localized brain injuries leading to delusional misidentification, a functional connectivity study has revealed connections between the brain lesions and regions involved in familiarity perception (left retrosplenial cortex) and belief evaluation (right prefrontal cortex) as possible correlates of the two factors [31].

To elucidate possible neural substrates of somatic delusions, we previously investigated structural brain alterations in patients with DI, using magnetic resonance imaging (MRI) and voxel-based as well as source-based morphometry techniques. In two studies, we compared patients with DI to healthy controls (HC) [32, 33], and recently, we compared patients with DI to HC and to patients presenting with non-somatic DD (NS-DD) [34]. Our results showed alterations in prefrontal, insular, striatal and thalamic brain regions of patients with DI when compared to healthy volunteers and to patients with NS-DD. Another structural imaging study of patients with SZ and somatic delusions has also shown structural abnormalities in frontal, insular and thalamic regions [35]. As discussed previously, alterations in those regions support the notion of impaired neural networks related to somatosensory perception and top-down regulation of sensory input, i. e. prediction processing [34]. Thus, impaired somatosensory networks could mediate somatic delusions as opposed to delusions without somatic content.

Given the prominent role of the cerebellum in somatosensory perception, the question arises whether cerebellar alterations might be involved in the development of delusions with somatic content. Here, we hypothesized that structural deficits in the cerebellum, especially in sensorimotor subregions, are associated with somatic delusions. In the present study, we therefore investigated GMV in patients with somatic and non-somatic monothematic delusions, diagnosed either as delusional disorder or as SZ, as well as in HC. For this purpose, we employed MRI and voxel-based morphometry (VBM) with cerebellum-optimized segmentation techniques. We predicted that somatosensory regions of the cerebellum would be differentially affected in patients presenting with somatic delusions, in contrast to individuals presenting with non-somatic content.

## Methods

### Participants

We analyzed data from 50 patients presenting with persistent monothematic delusions, diagnosed as DD or SZ (Table 1). All patients were cases observed and treated at the Psychiatric Department of the General Hospital Bruneck, South Tyrol, Italy. Diagnoses of DD or SZ according to DSM-IV-TR criteria were made by a board-certified psychiatrist (MH) based on a detailed clinical history and supporting clinical findings. The patient sample consisted of 14 individuals with S-DD, 18 individuals with NS-DD, and 18 individuals with SZ presenting with persistent non-somatic delusions. Additionally, a total of 32 HC were included.

The S-DD group consisted of 6 males and 8 females with a mean age of 72.6 (standard deviation [SD] = 9.3) years. This clinically homogeneous group presented with typical symptoms of “delusional infestation”, i. e. with fixed false beliefs that small living pathogens infested their body [23]. Mean disease duration was 6.9 years (SD = 10.0). Comorbid medical conditions were present in seven of these individuals, including hypertension and associated cerebral small vessel disease with typical white matter lesions, as ascertained by MRI (*n* = 5), struma nodosa with a history of radiotherapy (*n* = 1) and severe impairment of hearing and vision (*n* = 2). None of the patients fulfilled diagnostic criteria for SZ. All patients received antipsychotic treatment (mean chlorpromazine [CPZ] equivalents = 188.7, SD = 101.0 [36]).

The NS-DD group included 5 males and 13 females with a mean age of 55.9 (SD = 15.3) years. These patients presented with the following non-somatic delusional content: paranoid and persecutory ideas only (*n* = 9), mixed paranoid/poisoning (*n* = 3), jealousy (*n* = 2, without any association to any substance-use disorder), poverty (*n* = 2) or hypochondria (*n* = 2), where the latter did not report any delusional content related to being infested with particular pathogens. None of the patients fulfilled diagnostic criteria for an affective disorder or SZ. Mean disease duration was 13.9 years (SD = 11.1). In this group, 15 patients received antipsychotic treatment (mean CPZ equivalents in medicated patients = 221.4, SD = 153.1), and 3 patients were unmedicated.

The SZ group included 11 males and 7 females with a mean age of 48.3 (SD = 10.2) years. Those patients presented with the following persistent non-somatic delusional content: paranoid/persecutory (*n* = 14) or poisoning (*n* = 4). Mean disease duration was 19.7 years (SD = 10.2). All patients received antipsychotic treatment (mean CPZ equivalents = 443.5, SD = 180.0).

All medicated patients were under treatment with antipsychotics for at least 1 year prior to the MRI scan. None of the patients had a history of substance-use disorder or met criteria for major neurocognitive disorder.

The HC group consisted of 15 males and 17 females with a mean age of 57.4 (SD = 12.4) years. All HC were contacted by phone with the help of the local electronic hospital information system. A history of a mental disorder according to DSM-IV-TR criteria was ruled out by a board-certified psychiatrist (MH) by means of semi-structured interviews. None of the HC had a history of psychotropic drug treatment and no history of a major mental disorder in a first-degree relative. Further exclusion criteria for HC were a neurological history or a severe medical condition.

All patients and HC were right-handed, as identified by their dominant writing hand.

Out of all participants, the 14 S-DD patients, the 18 NS-DD patients and 12 HC had already been considered in a previous study, which primarily investigated cortical correlates of delusional disorder [34]. None of the SZ patients had been considered in previous studies.

To investigate differences in age, disease duration or CPZ equivalents, t-tests were used. Chi-squared tests were employed to test for gender differences between the groups. A nominal *p* < 0.05 was defined, uncorrected for multiple comparisons. Due to significant age differences between the patient groups, two age-matched HC groups were assigned to distinct patient groups. The HC group that was matched with S-DD individuals consisted of 9 males and 8 females with a mean age of 67.2 (SD = 8.7) years. A second group of HC that was matched with NS-DD and SZ patients comprised 12 males and 14 females with a mean age of 53.0 (SD = 9.2) years.

### Structural neuroimaging data acquisition and analysis

Structural data were acquired in the Department of Radiology at the General Hospital Bruneck, South Tyrol, Italy, using an MRI system at 1.0 Tesla (Philips INTERA, Release 11, Best, The Netherlands). The MRI parameters of the 3D T1 gradient echo recalled (fast field echo, FFE) sequence were as follows: TE = 6.9 ms; TR = 25 ms; FOV = 230 mm (AP), 172 mm (RL); resolution = 0.9 mm × 0.9 mm × 0.9 mm isotropic voxel with no slice gap; number of slices = 170.

Analyses were performed using Statistical Parametric Mapping (SPM) software package, version 12 [37] running on MathWorks MATLAB, version 2012a [38]. For data preprocessing, the Spatially Unbiased Infratentorial Template (SUIT) toolbox was used [39]. The advantages of SUIT for VBM are achieved through an improved overlap of cerebellar structures and by masking the image before reslicing it into SUIT space to ensure that no supratentorial gray matter can bias segmentation [40]. After visually checking for data artifacts and setting the image origin at the anterior commissure in each subject, infratentorial structures, i. e. cerebellum and brainstem, were isolated from the surrounding tissue. The isolation procedure includes the unified segmentation approach [41] as implemented in SPM, which segments the brain into tissue types, specifically gray and white matter, as well as cerebrospinal fluid. To exclude tissue outside the brainstem or cerebellum that was included by the isolation algorithm, we hand-corrected the isolated maps using the Caret software [42, 43]. Subsequently, GMV segments were normalized to the SUIT template, using the Diffeomorphic Anatomical Registration using Exponentiated Lie algebra (DARTEL) registration method [44]. To correct for volume changes through normalization, a modulation was applied using the Jacobian determinants of the deformation matrix. Prior to random-effects analyses between the groups, the modulated normalized GMV maps were smoothed using a full width at half maximum (FWHM) Gaussian kernel of 6 mm.

Between-group comparisons were computed using two-sample t-tests as implemented in SPM12. Seven models were computed. First, comparisons between the patient groups and matched HC groups were calculated. Next, comparisons between the patient groups were computed. Finally, to test for transnosologic effects, we computed a model that included the entire group of HC and all patients irrespective of diagnostic category and delusional content. Age, gender, CPZ equivalents and disease duration were included as nuisance variables in all analyses. An absolute threshold of 0.1 was used to prevent effects occurring at tissue border regions. Between-group differences were assessed using a significance threshold of *p* < 0.005 (uncorrected at the voxel level). Based on random field theory (RFT) [45], an empirically determined extent threshold according to the expected number of voxels per cluster within the respective contrast was employed. To determine anatomical locations of the peak voxels of significant clusters emerging from between-group comparisons we used the SPM Anatomy Toolbox v2.1 [46], which includes a probabilistic atlas of the human cerebellum [47].

## Results

### Demographic and clinical data

S-DD patients and their HC group did not significantly differ with respect to age and gender (*p* = 0.10 and 0.32, respectively). But S-DD patients were significantly older compared to NS-DD and SZ patients (*p* = 0.001 and 0.0001, respectively). S-DD patients did not significantly differ from NS-DD or SZ patients with respect to gender (*p* = 0.15 and *p* = 0.12, respectively). S-DD and SZ patients significantly differed with respect to disease duration (p = 0.001) and CPZ equivalents (*p* = 0.00001). S-DD and NS-DD patients did not significantly differ with respect to disease duration (*p* = 0.07) and CPZ equivalents (*p* = 0.050).

NS-DD and SZ patients did not significantly differ from HC with respect to age (*p* = 0.44 and 0.12, respectively). NS-DD and SZ patients also did not significantly differ with respect to age (*p* = 0.09). NS-DD patients significantly differed from HC and SZ with respect to gender (*p* = 0.04 and 0.004, respectively). SZ patients and HC did not significantly differ with respect to gender (*p* = 0.06). NS-DD and SZ patients did not differ significantly with respect to disease duration (*p* = 0.11), but they differed significantly with respect to CPZ equivalents (*p* = 0.0003).

### Cerebellar gray matter volume differences

**Fig. 1:**
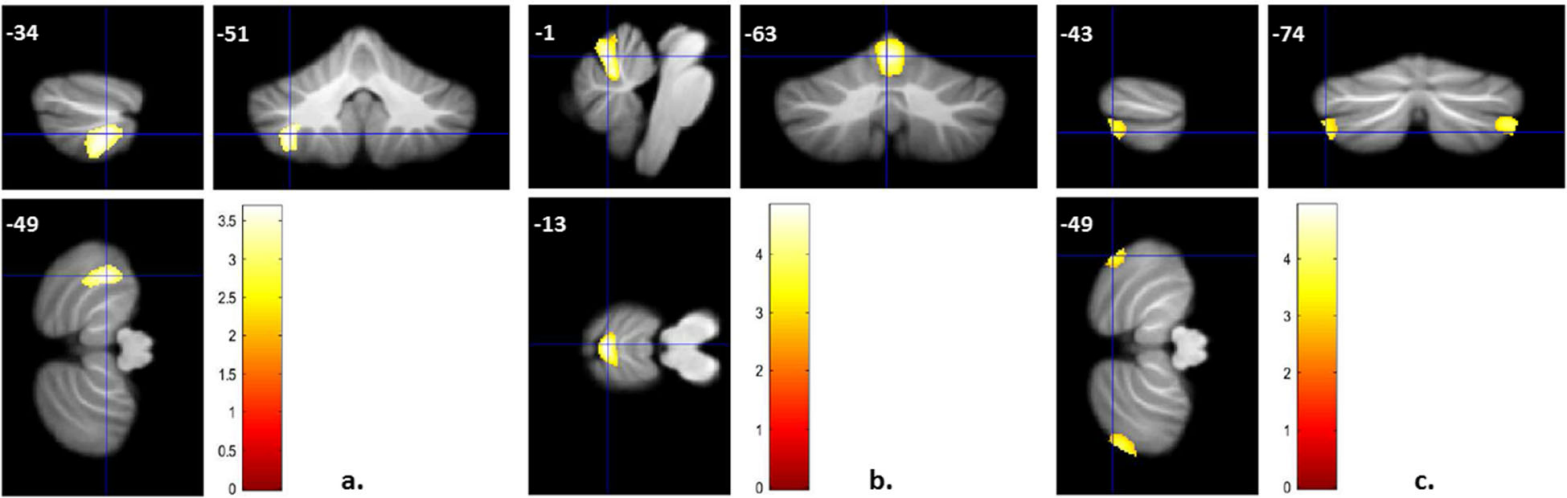
**a** Decreased lobule VIIIa GMV in patients with S-DD compared to HC. *p* < 0.005 uncorrected for height, k > 602 voxels. **b** Decreased lobule V GMV in S-DD compared to NS-DD patients. *p* < 0.005 uncorrected for height, k > 505 voxels. **c** Increased lobule VIIa/crus II GMV in S-DD compared to NS-DD patients. *p* < 0.005 uncorrected for height, k > 505 voxels. Shown are results of two-sample t-tests, adjusted for age, gender, CPZ equivalents and disease duration. The color bars represent T-values

In analyses that considered the entire cerebellum, lower GMV in S-DD patients compared to age-matched HC was found in left lobule VIIIa (x = − 34, y = − 51, z = − 49, Z = 3.25, 74% probability of belonging to this specific cerebellar region, k = 1635 voxels); see Fig. 1a. Regions with increased GMV in S-DD patients compared to HC were not found. Compared to NS-DD patients, S-DD individuals showed decreased GMV in lobule V (x = − 1, y = − 63, z = − 13, Z = 4.04, 58% probability, k = 1724 voxels); see Fig. 1b. In addition, increased GMV in S-DD compared to NS-DD patients was found in bilateral lobule VIIa/crus II (x = − 43, y = − 74, z = − 49, Z = 3.29, probabilities 68–100%, k = 558 voxels and x = − 34, y = − 88, z = − 36, Z = 4.10, probabilities 61–85%, k = 1309 voxels); see Fig. 1c. Compared to SZ, S-DD patients showed decreased GMV in lobule V (x = 6, y = − 63, z = − 7, Z = 3.71, 79% probability, k = 491 voxels), yet this effect did not survive cluster-correction.

**Table 1:**
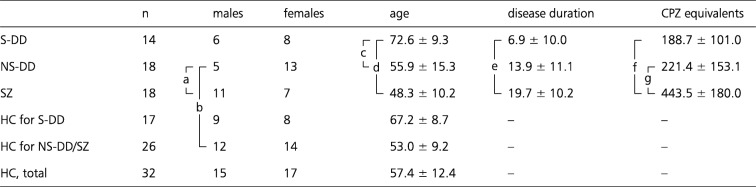
Demographic and clinical characteristics

Values are given as mean ± standard deviation. Vertical brackets indicate significant differences between groups (a and b refer to gender differences). The respective *p*-values are: (a) 0.004, (b) 0.04, (c) 0.001, (d) 0.0001, (e) 0.001, (f) 0.00001, (g) 0.0003

NS-DD patients did not exhibit significant GMV differences compared to HC or compared to SZ.

**Fig. 2:**
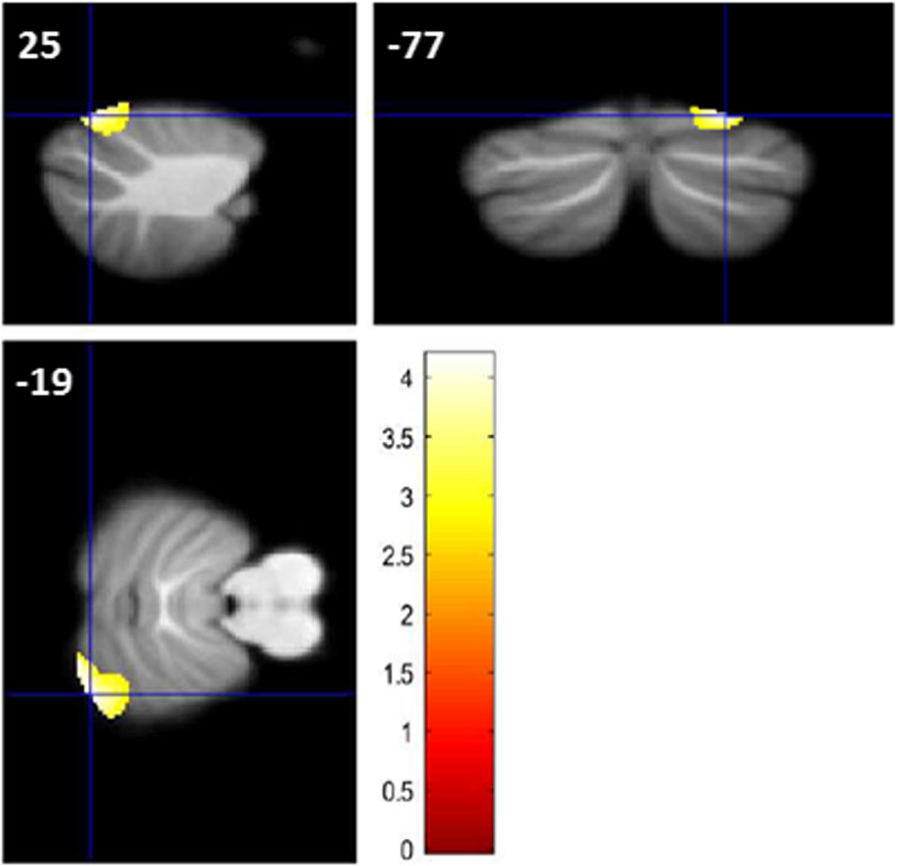
Increased lobule VIIa/crus I GMV in patients with SZ compared to HC. *p* < 0.005 uncorrected for height, k > 536 voxels. Shown are results of two-sample t-tests, adjusted for age, gender, CPZ equivalents and disease duration. The color bar represents T-values

Compared to HC, SZ patients showed increased GMV in a cluster comprising right lobules VI and VIIa/crus I (x = 25, y = − 77, z = − 19, Z = 3.78 and x = 18, y = − 80, z = − 19, Z = 3.77, k = 852, with probabilities of belonging to lobule lobules VI or VIIa/ccrus I of 47 and 62%, respectively; see Fig. 2.

The model that included the entire HC and patient samples did not yield significant findings.

## Discussion

In this structural MRI study, we employed optimized segmentation techniques to investigate cerebellar GMV in patients with SZ and DD presenting with specific monothematic delusional content, i. e. somatic vs. non-somatic delusions. Three main findings emerged: First, comparing S-DD patients to HC, we could detect lower GMV in left lobule VIIIa. Second, comparing S-DD to NS-DD patients, we could detect lower GMV in lobule V and higher GMV in bilateral lobule VIIa/crus II. It is noteworthy that comparing S-DD and SZ patients, a lower GMV in lobule V was also found, even though this difference was not significant. Third, in individuals with SZ, GMV alterations could also be identified, yet those showed a distinct pattern that differed from HC and patients with S-DD.

Recent research has established that at least three functional domains of the cerebellum can be distinguished, i. e. sensorimotor and multimodal cognitive/affective subdivisions. Sensorimotor functions of the cerebellum are localized in the anterior lobe (i. e. lobule I–V) and lobule VIII, while cognitive and affective functions are mainly localized in the posterior lobe (i. e. lobules VI–IX), with lobule VI constituting a transition zone. This is evidenced by functional imaging studies in humans, as well as tract-tracing experiments in animals [2, 3]. Accordingly, the subregions that showed lower GMV in S-DD patients (i. e. lobules V and VIII) pertain to sensorimotor regions, while the subregions that showed higher GMV in S-DD patients (i. e. lobule VIIa/crus II) and in SZ patients (i. e. lobules VI and VIIa/crus I) pertain to cognitive/emotional regions.

Thus, our finding of impaired GMV in cerebellar lobules V and VIII in S-DD patients supports the hypothesis of an association between specific deficits in sensorimotor regions of the cerebellum and delusional themes with somatic content. Our findings are in line with previous structural whole-brain imaging studies, which have already identified several cerebral loci with GMV alterations specific to patients with somatic delusions. As already mentioned, previous studies of S-DD patients have shown impaired GMV in prefrontal, insular, striatal and thalamic brain regions [32–34] and another study of SZ patients with somatic delusions has shown GMV alterations in frontal, insular and thalamic regions [35]. The dorsal striatum and the insula are known to be part of the peripersonal space network, which integrates visual and tactile perceptions and maintains an internal representation of the space near the body surface [48]. The putamen is also involved in prediction of sensory perception [49] and the insula in prediction of interoception [50], including pain and disgust [51]. Thus, similar to our findings of alterations in sensorimotor regions of the cerebellum, alterations in those cerebral regions also support the notion of impaired neural networks related to somatosensory perception and top-down regulation of sensory input [34]. The aforementioned studies however have not robustly identified cerebellar alterations. In our previous study utilizing whole-brain VBM, we found lower GMV in left lobule VIIa/crus I when comparing S-DD to HC, but no difference when comparing S-DD to NS-DD [34]. The lack of cerebellar findings in previous studies might be due to the employed whole-brain morphometry methods: In particular, VBM methods using conventional whole-brain templates for data normalization can lead to poor alignment of cerebellar subregions [40]. The SUIT toolbox employed in the present study provides a high-resolution, spatially unbiased template of the human cerebellum. The cerebellar template preserves anatomical detail of cerebellar subregions using automated nonlinear normalization methods, thus achieving a more accurate intersubject-alignment compared with whole-brain methods. Accordingly, SUIT has been shown to be more sensitive to cerebellar change compared with conventional whole-brain VBM in some cases [40, 52].

Our findings of GMV alterations in cognitive/emotional regions of the cerebellum in S-DD and SZ patients are in line with the concept of cognitive dysmetria in psychotic disorders [9] and with the observation of psychotic symptoms in patients with posterior lobe lesions [3]. They are also in line with our previous study linking reduced GMV in lobules VIIb and VIIIa in SZ patients with persistent AVH [17]. In that study, a negative correlation of GMV with global positive symptoms and thought disturbance scores (PANSS-P and BPRS-THD) could be detected, but no correlation with AVH-specific scores. Those results suggest that GMV impairments in cognitive/emotional regions of the cerebellum are involved in the emergence of positive symptoms in general, but not exclusively in the emergence of AVH. If deficits in cognitive/emotional regions of the cerebellum are indeed involved with the emergence of different symptoms in psychotic disorders, it seems likely that somatic delusions are not only associated with alterations in sensorimotor regions, but with alterations in cognitive/emotional regions as well. However, it cannot be derived from our structural data alone whether both alterations in sensorimotor and cognitive/emotional regions detected in S-DD patients are related, e. g. by contributing to a single process or by contributing to distinct but interacting processes.

The notion of impaired neural networks related to somatosensory perception and prediction in patients with somatic delusions clearly matches the two-factor model of delusions, where a content-specific first factor is responsible for the emergence of delusional ideas [27]. In the case of somatic delusions, abnormal somatic perceptions or erroneous mismatches of somatic perceptions and predictions could constitute that factor. Abnormal sensations could then lead to the idea of infestation as a possible explanation for those sensations by abductive inference. According to the model, a second factor would also be required that is responsible for impaired reasoning and failure to reject the implausible idea of infestation. That second factor would not be specific to the delusional content and is not addressed by the present study, though alterations of Lobule VIIa, as detected in this study, may be suggestive of disrupted corticocerebellar control. Neuropsychological studies suggest the right dorsolateral prefrontal cortex to play a critical role in belief evaluation, and an impairment in this area would be expected [53]. Whether the reported alterations in cognitive/emotional regions of the cerebellum might also contribute to impaired belief evaluation cannot be inferred from our structural data and therefore needs to be specifically addressed by (task-based) functional neuroimaging studies.

This study has several limitations. One limitation is the relatively small sample size. In this regard, it is worth noting that poor illness insight is very frequent in delusional disorders. As this hampers the acquisition of larger sample sizes, it is not surprising to see modest sample sizes in the extant literature as well. Limitations of this study also include possible effects of group differences, especially regarding age, gender, medication and disease duration. To account for the large age differences, distinct HC groups were defined, and we included age, gender, CPZ equivalents and disease duration as nuisance variables in our analysis. It is worth noting that S-DD patients typically feature a rather late disease onset (mean age at diagnosis of 61.4 years in a population-based study [54]), which explains the higher mean age in this group. As we focused on differences between somatic and non-somatic delusional contents in this study, no other symptom dimensions were assessed, like overall symptom severity or other specific aspects of delusions. We thus cannot rule out that other unknown factors might have influenced our findings. In this context, we also acknowledge heterogeneous comorbid medical conditions, especially in the S-DD sample. Regarding delusional content, we chose to only include S-DD patients with delusional infestation to keep the S-DD group as homogeneous as possible. Thus, the important question remains whether our findings are specific to delusional infestation or apply to S-DD in general. It seems plausible that other types of S-DD, especially delusional ideas related to hypochondric fears, but also ideas related to internal organs as opposed to the body surface, are associated with different neural defects. On the other hand, delusional content in participants with non-somatic delusions was heterogeneous, which impedes further inference about content-specific alterations in those individuals. A comparison of more homogenous groups would be desirable for future studies. As another limitation, the relatively low MRI field strength (1.0 T) employed in this study may have prevented us from detecting more subtle cerebellar alterations between the patient groups, or between patients and HC. We also acknowledge that reflections on functional mechanisms underlying the emergence of delusions are speculative, because the present study includes structural data only. Future studies are warranted that combine structural and functional imaging, to specifically investigate corticocerebellar connectivity regarding impaired cerebellar regions. Finally, the lack of correction for multiple comparisons after seven pair-wise t-tests is another limitation of this study. Given the relatively modest sample size and the scanner field strength, a conservative correction (e. g. Bonferroni correction) for multiple comparisons would have very likely increased the probability of false negative findings. We acknowledge the difficulties of this trade-off. Yet at the same time, considering our a-priori hypothesis of aberrant sensorimotor subregions, the neurobiological plausibility of our findings further supports their validity.

## Conclusions

We could confirm cerebellar deficits across distinct psychotic disorders, thus highlighting the role of the cerebellum across diagnostic categories. In addition, our data suggest possible content-related neural signatures of cerebellar dysfunction in individuals presenting with somatic delusions in contrast to those exhibiting non-somatic delusional beliefs. Future multimodal neuroimaging research needs to take into account putative functional consequences of cerebellar volume loss with respect to corticocerebellar connectivity in distinct neural systems that may promote specific delusional content.

## Data Availability

The data that support the findings of this study are available on reasonable request from the first and senior authors (MH and RCW). Raw data are not publicly available due to regulations and directives of the Health District Bruneck.

## Abbreviations

AVH: Auditory verbal hallucinations
BPRS-THD: Brief psychiatric rating scale, thought disturbance score
CCTCC: Cortico-cerebellar-thalamic-cortical circuit
CPZ: Chlorpromazine
DARTEL: Diffeomorphic anatomical registration using exponentiated lie algebra
DI: Delusional infestation
FWHM: Full width at half maximum
GMV: Gray matter volume
HC: Healthy controls
k: Cluster extent threshold
MRI: Magnetic resonance imaging
NS-DD: Non-somatic delusional disorder
PANSS-P: Positive and negative syndrome scale, positive score
RFT: Random field theory
SD: Standard deviation
S-DD: Somatic delusional disorder
SUIT: Spatially unbiased infratentorial template toolbox
SZ: Schizophrenia
VBM: Voxel-based morphometry
